# Supplementary material for: Prebiotic Xylo-Oligosaccharides Ameliorate High-Fat-Diet-Induced Hepatic Steatosis in Rats
Source: Nutrients. 2020 Oct 22;12(11):3225. doi: 10.3390/nu12113225 (PMC7690286; doi:10.3390/nu12113225)
Supplement: Supplementary file 1 [file nutrients-12-03225-s001.pdf]

SUPPLEMENTARY METHDOS

Histopathologic grading of the proximal colon

The grading was based on the prevalent constellation of changes detected over all (4-6 per rat) sections.

Surface epithelial injury:

Grade 1 (minimal to mild). Epithelial cells show focal to multifocal degenerative changes: increased eosinophilia, attenuation (diminished height) and irregular palisading (dysplasia).
Grade 2 (moderate). Surface epithelium exhibits widespread and more pronounced degenerative changes; eosinophilia, vacuolation and stacking, in comparison to grade 1. Epithelium also shows focal desquamation and apoptosis.

Goblet cell abundancy (hyperplasia):

Grade 0. Goblet cells reside generally in the basal half of crypts. Only single goblet cells are present in the surface epithelium.
Grade 1. Goblet cells extend over half crypt height and some extend to neck area. Some goblet cells are present in the surface epithelium.
Grade 2. Goblet cell extend consistently over half crypt height. Goblet cells are abundantly present in the surface epithelium.

Crypt length and (distortion):

Grade 0. Crypts are of equal height and less than 25 cells high.
Grade 1. Crypts are multifocally over 25 cells high and/or mildly tortuous or show open lumens.

Mucosal leucocytes:

Grade 0. Crypts are on average separated by less than five lymphocytes and single macrophages, and there are less than 10 mucosal mast cells (globular leucocytes) / HPF. Single eosinophils but generally no neutrophils are present. Surface and apical crypt epithelium contains only single lymphocytes or mast cells (globular leucocytes).
There are less than xx lymphocytic aggregates? (not included into current grading).
Lymphocytic aggregate (cryptopatch). Rounded collection of closely packed mononuclear cells (mostly lymphocytes) approximately 50-100 µm in diameter, span two crypts. No germinal centre.
Grade 1 (minimal). Apical lamina propria contains focally to multifocally mildly elevated number of lymphocytes and small number of macrophages. Number of lymphocytes and globular leucocytes may be focally mildly increased in the surface/and or apical crypt epithelium.
Grade 2 (mild to moderate). Apical lamina propria contains multifocally to diffusely mildly to moderately elevated number of lymphocytes and small number of macrophages. Number of lymphocytes and globular leucocytes is generally mildly increased in the

surface/and or apical crypt epithelium and monocyte infiltrates spread basally among crypts.

Neutrophil and eosinophil numbers appeared to be unaffected by the dietary treatments.

SUPPLEMENTARY FIGURES AND RESULTS

S1

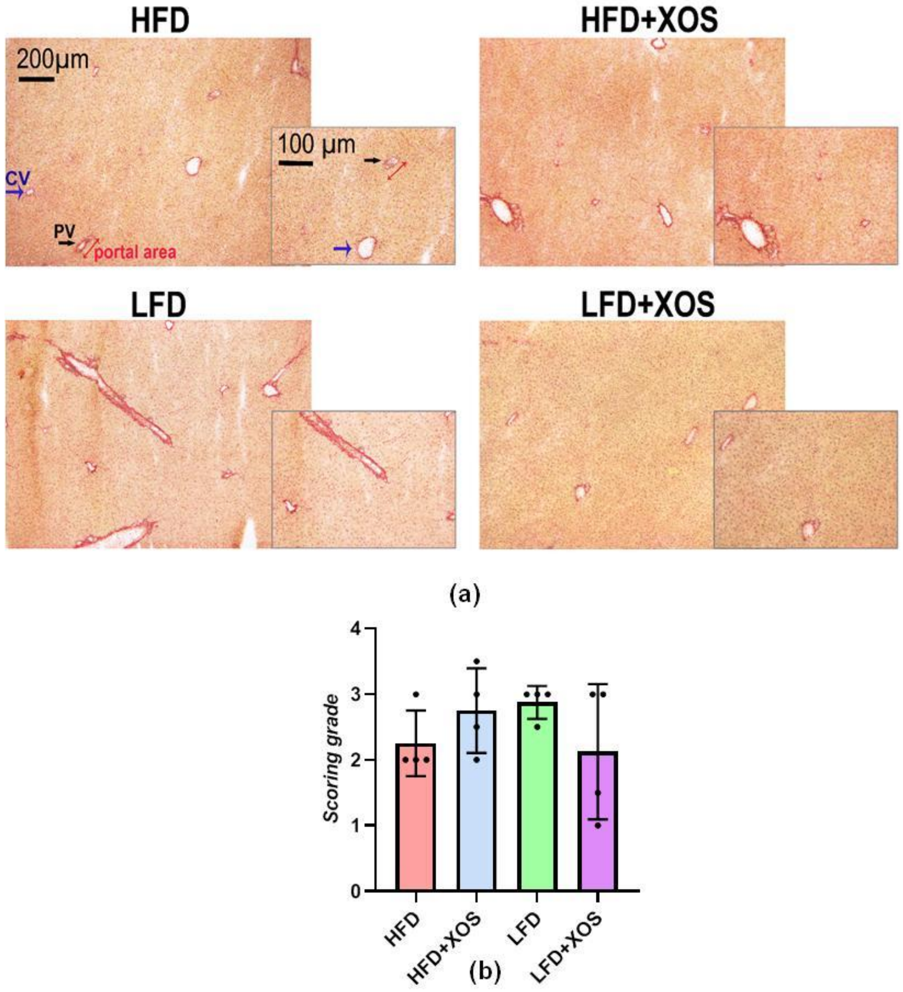

Supplementary figure S1. The Sirius Red staining for the estimation of the amount of liver fibrosis. **(a)** The histological samples of the medial lobe of the liver were cut with cryostat, and 10 μm sections were fixed with 4% paraformaldehyde and then stained with Weigert's hematoxylin. Finally, they were stained with Sirius Red and coverslipped with DePex following clearing in xylene. The stained sections were scanned with NanoZoomer microscope (Hamamatsu, Japan), and a blinded experimenter scored them; **(b)** As shown in the graph, no difference was found between the groups in the amount of fibrosis. Yet, there was at some extent fibrous expansion in portal areas and short fibrous septa in all groups. The graph shows the average scoring of four samples per group. The dots indicate different data points. Blue arrows indicate central vein (CV) and black arrows portal vein (PV).

A

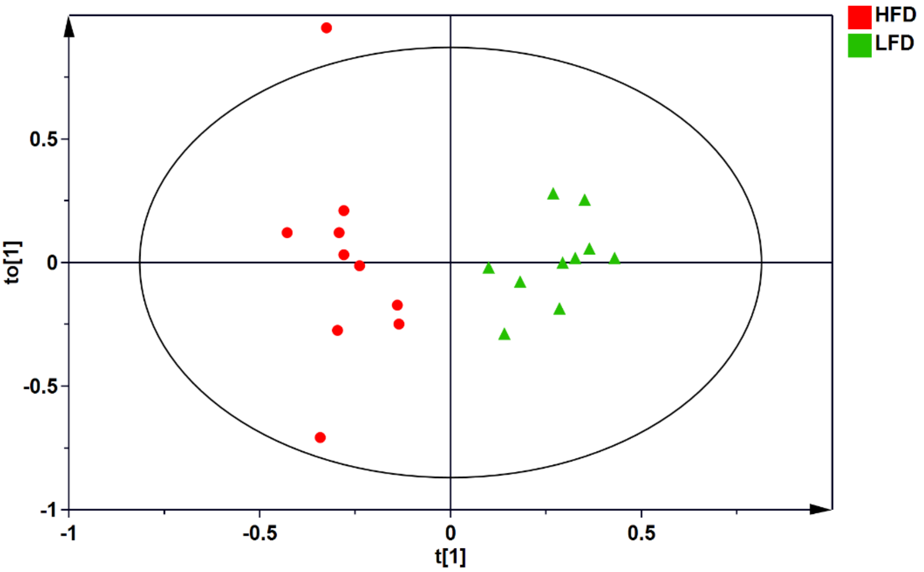

B

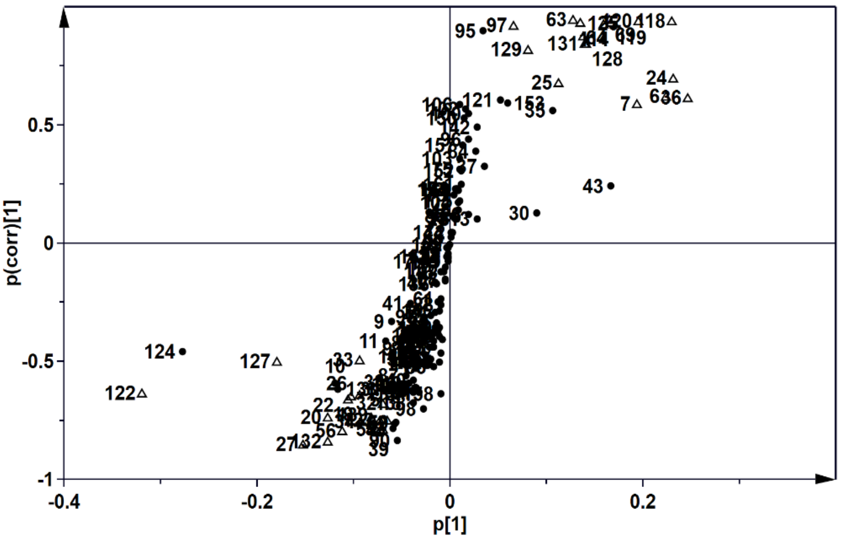

Supplementary figure S2. OPLS-DA of caecal metabolic profiles between the HFD and LFD. **A**) Score scatter plot; **B**) *S*-loading plot. Bin labels of the metabolites identified to be discriminatory are symbolized with empty triangles (Δ). Please refer to Supplementary table 1 to interpret the numbers of discriminatory bins. $R^2X_{(cum)}$ = 0.552 and $R^2Y_{(cum)}$ = 0.9, Q^2 = 0.841.

S3

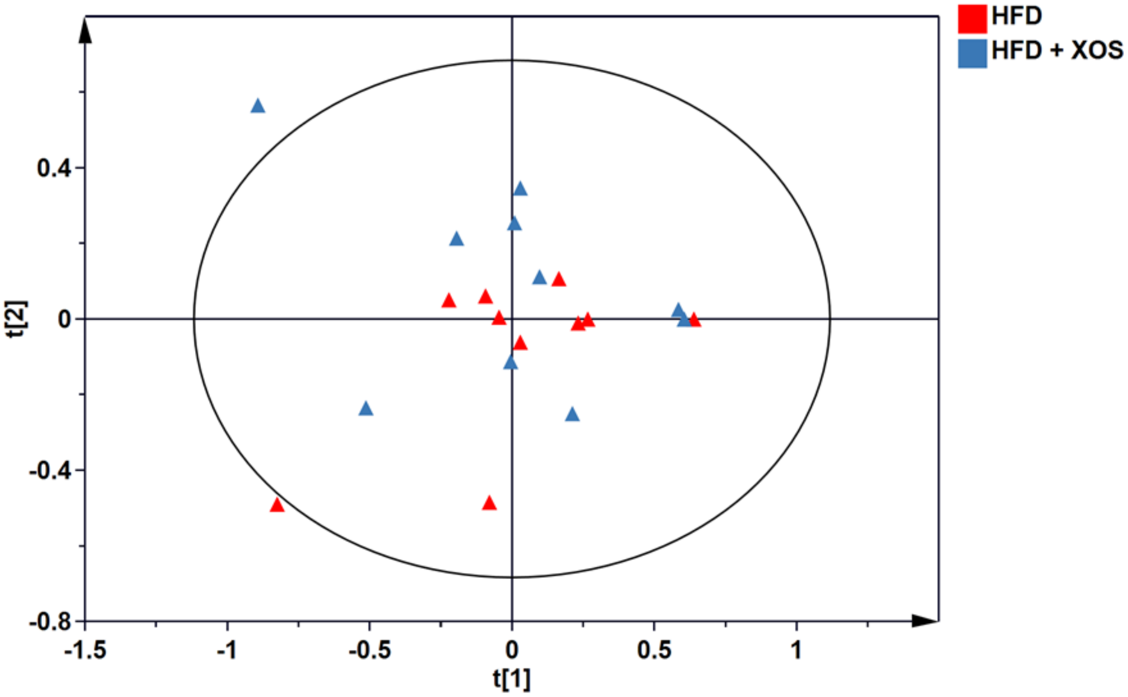

Supplementary figure S3. Score scatter plot of the Principal Component Analysis of the caecal metabolic profiles between the HFD and HFD+XOS rats.

S4 **A**

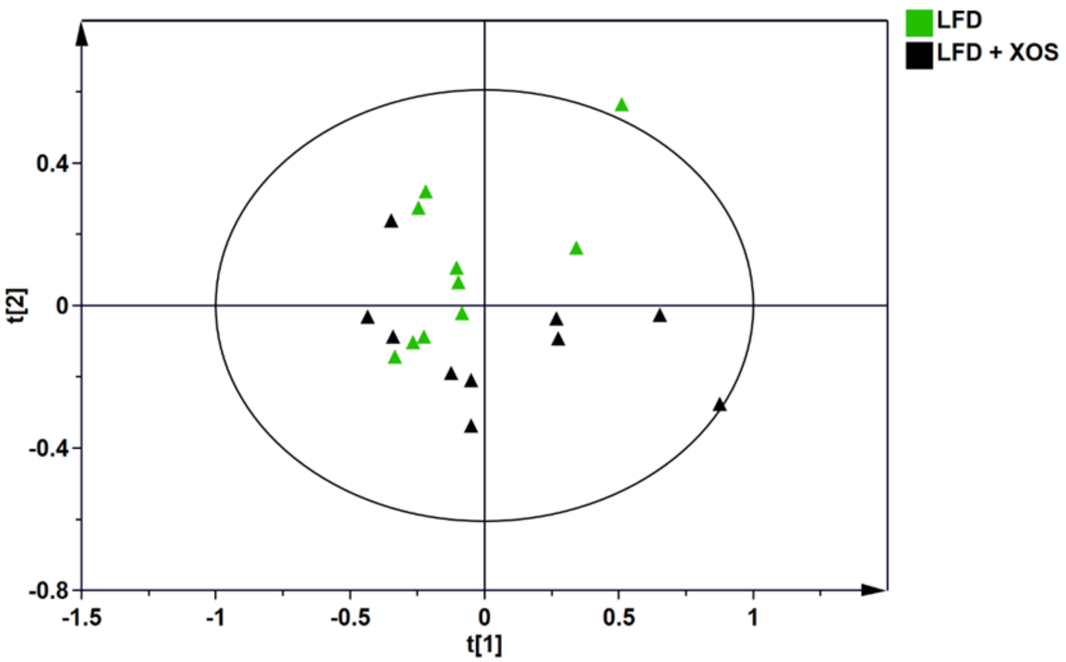

B

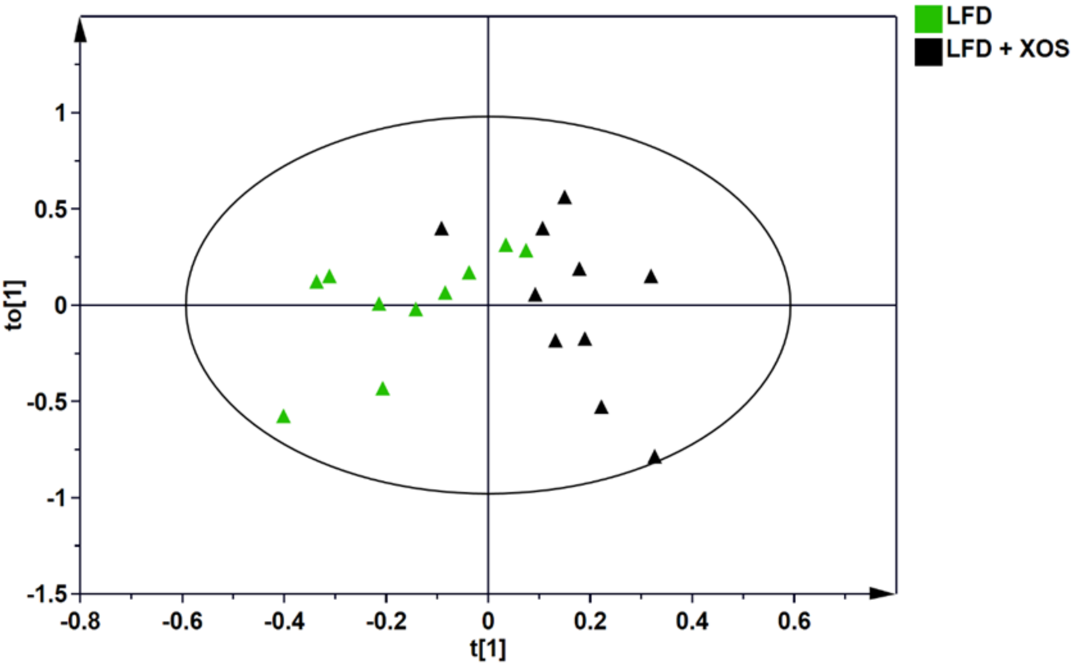

Supplementary figure S4. Multivariate analysis of the caecal metabolic profiles between the LFD and LFD+XOS rats. **A**) PCA Score scatter plot; **B**) OPLS-DA Score scatter plot. A1+1+0 component model OPLS-DA showed a kind of separation of the two groups based on component t[1]. However, the model diagnostics represented only a moderate goodness of fit (R^2X(cum) = 0.593 and R^2Y(cum) = 0.595), and a low predictive ability (Q^2 = 0.244).

S5

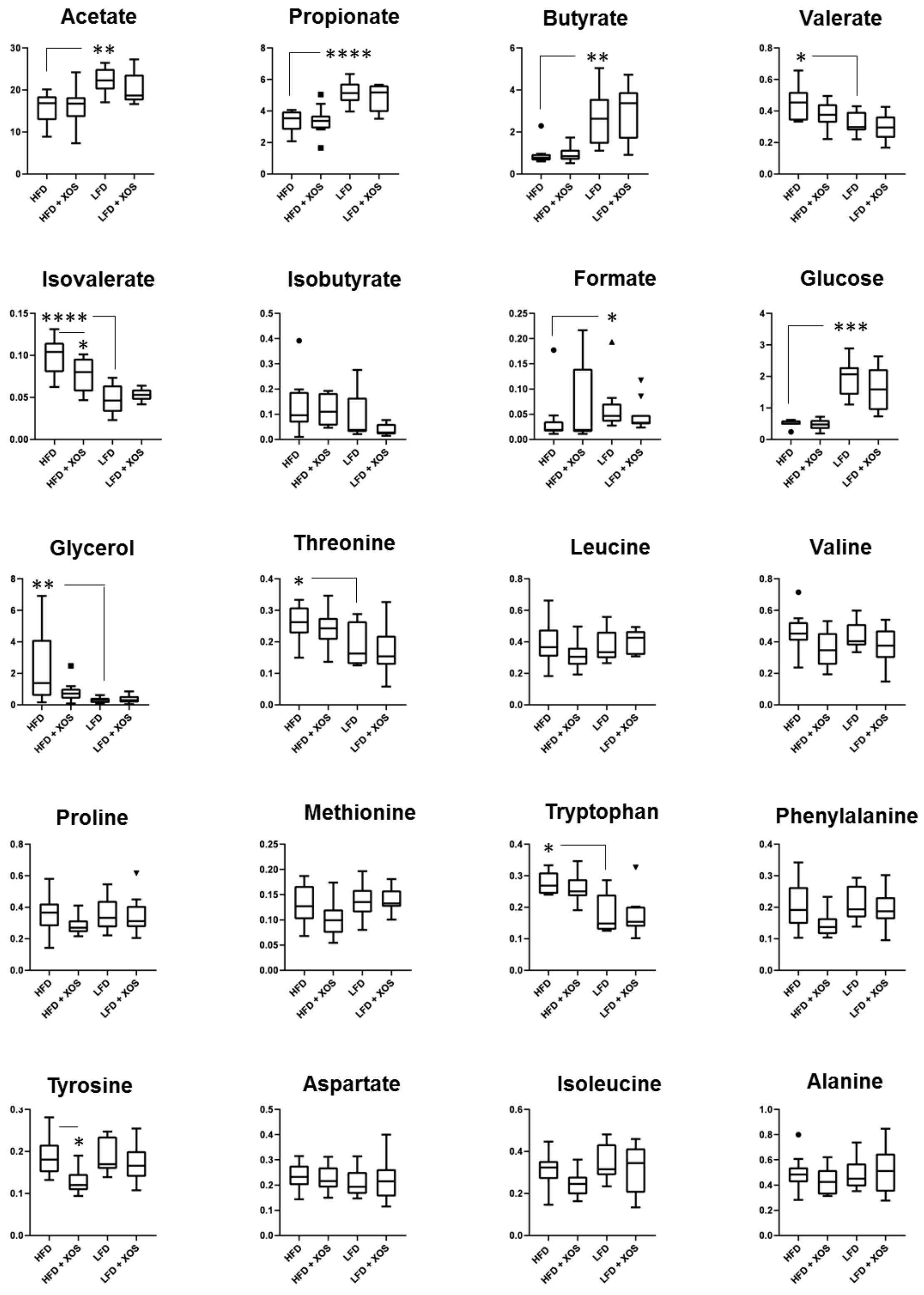
Acetate
Propionate
Butyrate
Valerate
Isovalerate
Isobutyrate
Formate
Glucose
Glycerol
Threonine
Leucine
Valine
Proline
Methionine
Tryptophan
Phenylalanine
Tyrosine
Aspartate
Isoleucine
Alanine
HFD
HFD + XOS
LFD
LFD + XOS

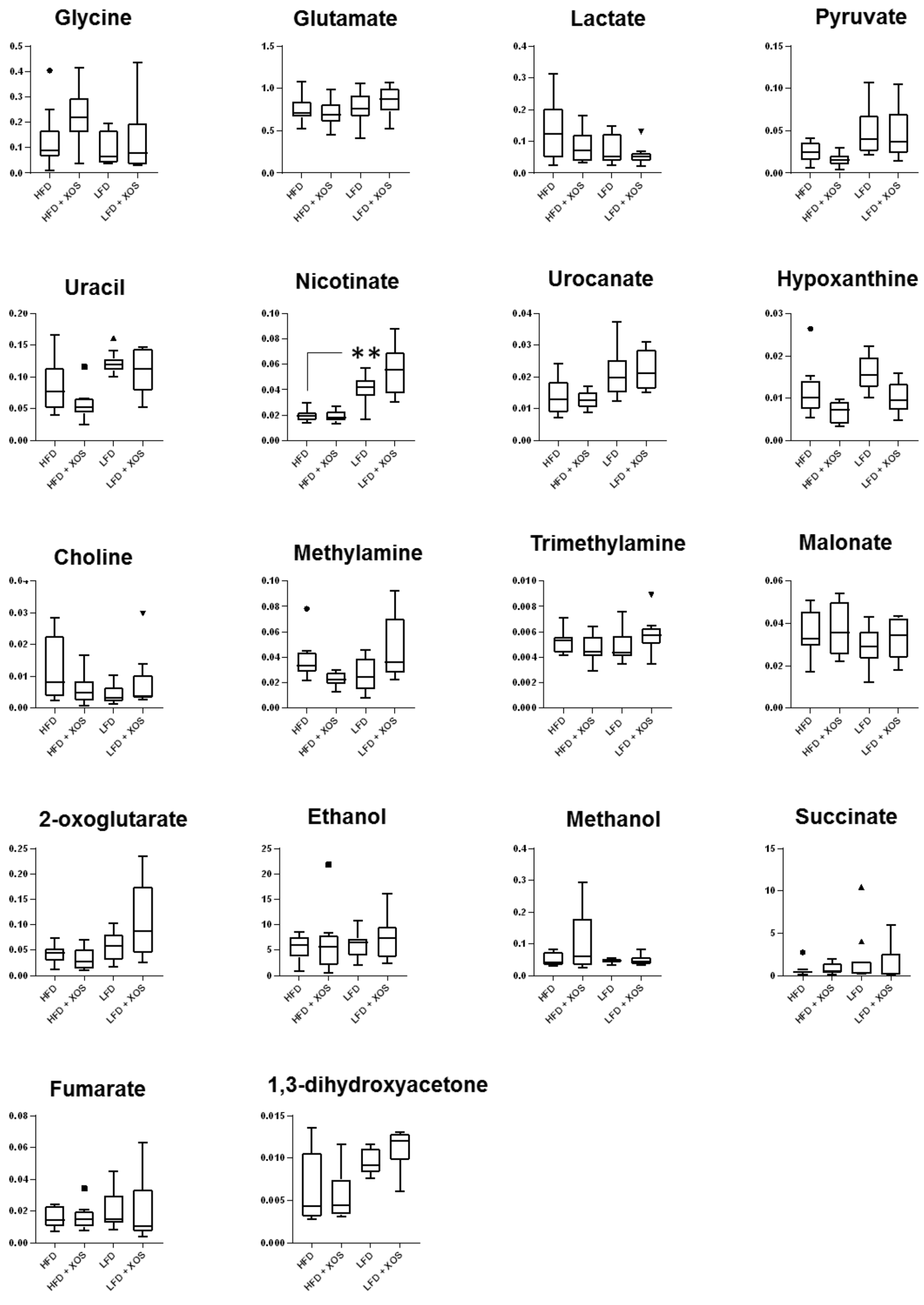

Supplementary figure S5. The concentrations of the caecal metabolites in different diet groups. The concentrations and differences were determined using Profiler module of Chenomx NMR Suite Professional 8.3. All concentrations are mmol/L. * $p < 0.05$, ** $p < 0.01$, *** $p < 0.005$, **** $p < 0.001$.

S6

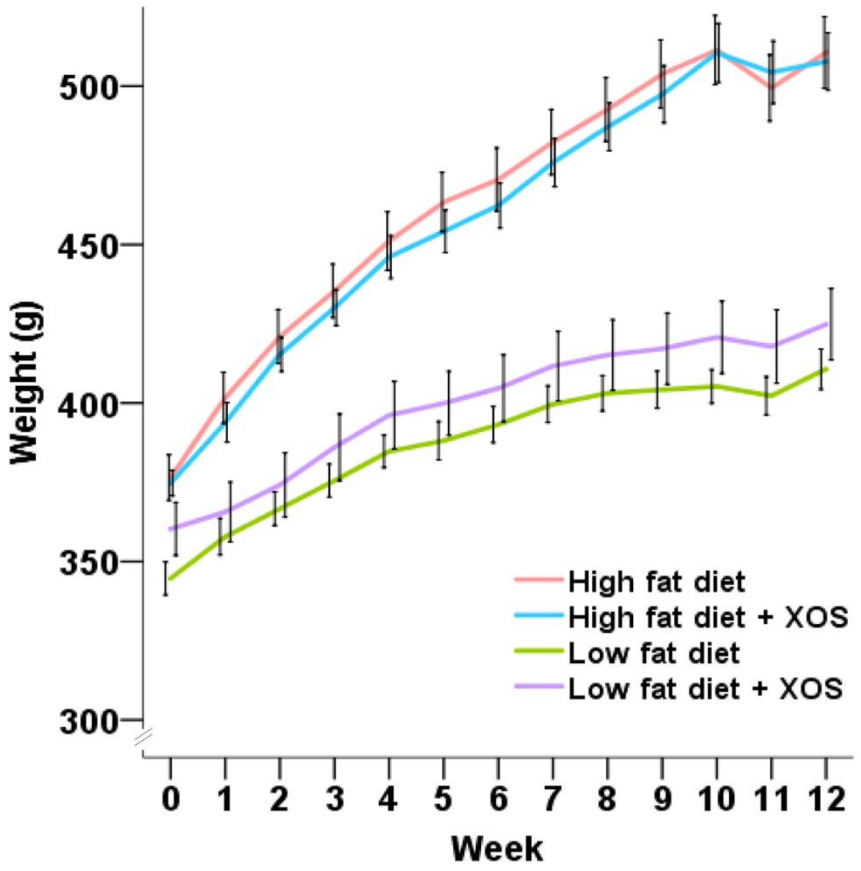

Supplementary figure S6. Development of body weight during the diet intervention. Body weight differed between the HFD and LFD groups during the diet intervention, but XOS supplementation did not affect the weight. The time point 0 indicates the time when the intervention started. Week (F (12, 72.76) = 68.99, p < 0.001), Diet (F (1, 446.9) = 807.1, p < 0.001), and Week * Diet (F (12, 72.76) = 8.54, p < 0.001).

S7

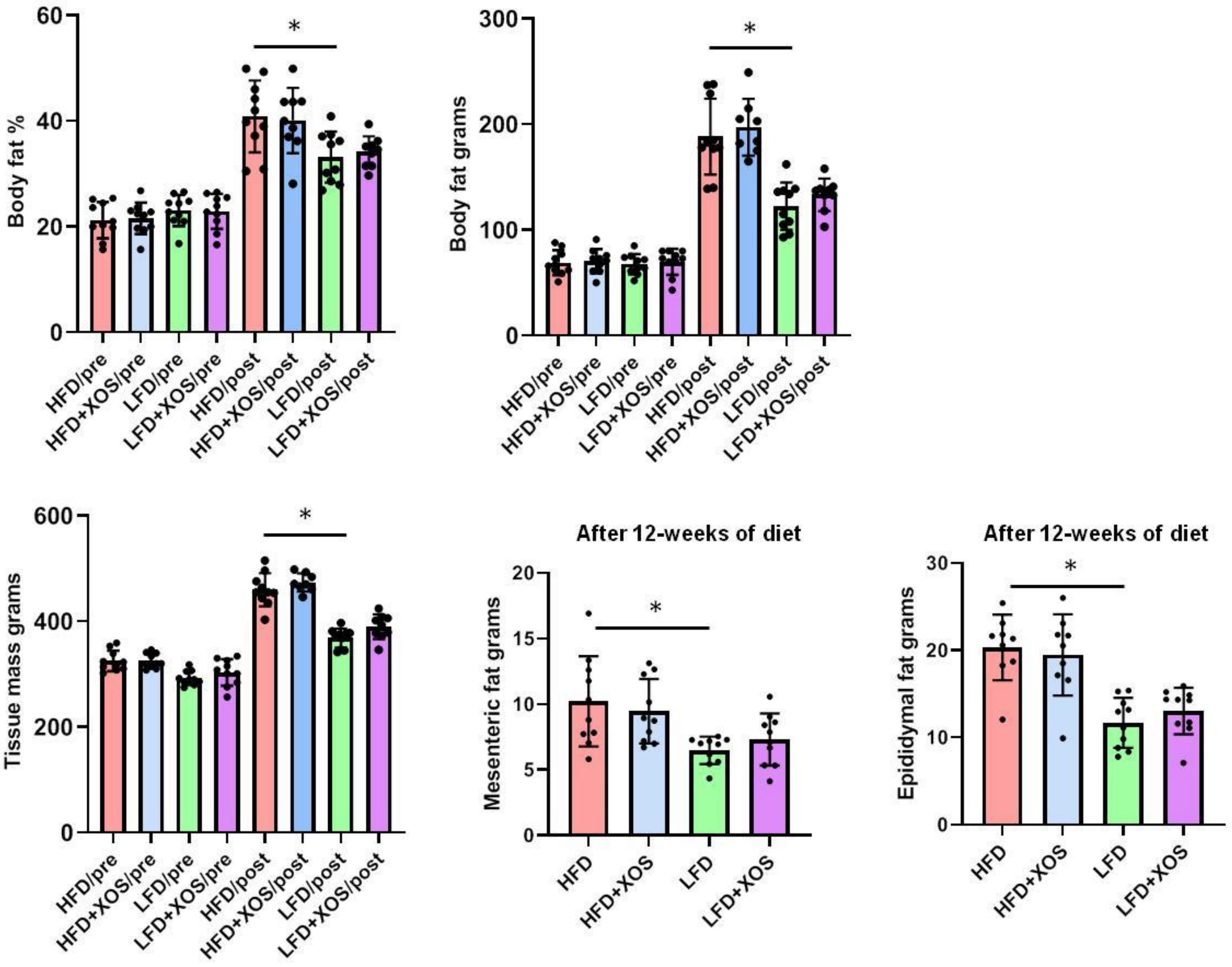

Supplementary figure S7. XOS did not modulate body composition measured by dual energy x-ray absorption or tissue weight at necropsy. Pre= before the diet intervention; post= after 12-weeks of diet. * denotes statistically significant difference between the groups that are connected with a line.

S8

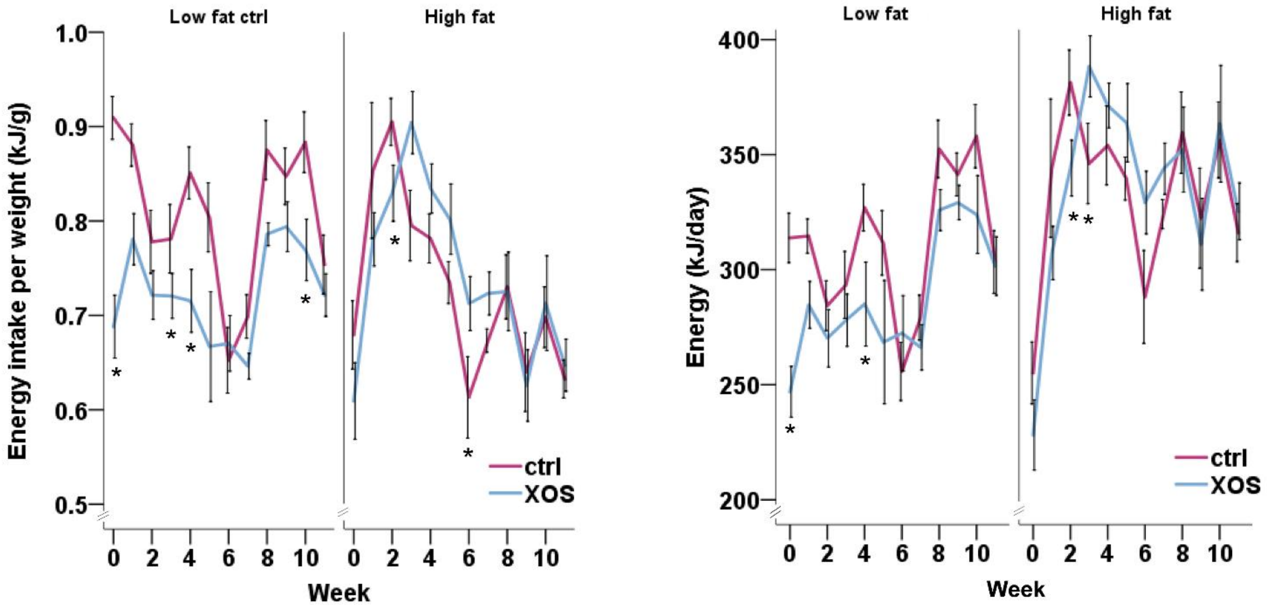

Supplementary figure S8. The energy intake during the diet intervention. The left graph shows the energy intake relative to weight (KJ/g) and the right graph the energy intake per day (KJ/day). * = significant difference between the groups (control vs. XOS). The statistically significant differences were calculated with linear mixed model ANOVA accounting for repeated measures for each individual.

Supplementary table 1. List of highly influential variables (bins) identified by combining *S*-plot, column loading plot and VIP values ≥ 0.7, as obtained from the OPLS-DA.

Bin no.	Chemical shift region (ppm)	Metabolite ID	VIP score
5	0. 8405–0.8595	Bile	≥ 1.0
6	0.8605–0.8845	Butyrate + valerate	≥ 1.0
7	0.8855–0.9095	Butyrate + isovalerate	≥ 1.0
19	1.250–1.30	Valerate	0.7 – 0.99
20	1.30–1.34	Threonine + lactate	≥ 1.0
24	1.523–1.56	Butyrate	≥ 1.0
25	1.56–1.59	Butyrate	≥ 1.0
26	1.60–1.68	Leucine	0.7 – 0.99
27	1.68–1.76	Leucine	≥ 1.0
34	2.08–2.12	Glutamate/methionine	0.7 – 0.99
35	2.12–2.14	Butyrate/methionine	≥ 1.0
36	2.14–2.185	Propionate	≥ 1.0
37	2.185–2.202	Propionate	0.7 – 0.99
38	2.202–2.245	Valine	≥ 1.0
39	2.245–2.2940	Valine	0.7 – 0.99
63	4.60–4.64	Glucose	≥ 1.0
64	4.64–4.678	Glucose	≥ 1.0
69	5.21–5.25	Glucose	≥ 1.0
114	3.215–3.265	Glucose	≥ 1.0
118	3.3650–3.4320	Glucose/proline	≥ 1.0
119	3.4320–3.4650	Glucose	≥ 1.0
120	3.4650–3.5050	Glucose	≥ 1.0
122	3.5320–3.5760	Glycerol	≥ 1.0

123	3.5880–3.610	Valine/threonine	0.7 – 0.99
124	3.620–3.6820	Glycerol/ethanol	≥ 1.0
125	3.6840–3.7180	Glucose	≥ 1.0
127	3.7550–3.802	Glycerol	≥ 1.0
128	3.802–3.840	Glucose	≥ 1.0
129	3.840–3.86	Glucose	0.7 – 0.99
131	3.874–3.910	Glucose	≥ 1.0
132	3.91–3.95	Unknown	≥ 1.0
139	4.206–4.2560	Threonine	0.7 – 0.99
97	7.56–7.60	Unknown	≥ 1.0

Supplementary table 2. Concentrations of the caecal metabolites that discriminated between the HFD and LFD. The values and differences were calculated using Profiler module of Chenomx NMR Suite Professional 8.3. The highlighted cells denote up-regulation of the metabolites. The values are expressed as mM (Mean ± SEM)

**Metabolite**	**HFD (Mean ± SEM)**	**LFD (Mean ± SEM)**	***p*-value (t-test)**
Acetate	16 ± 1.2	22 ± 0.9	0.0004 ***
Butyrate	0.91 ± 0.16	2.6 ± 0.39	0.0001 ***
Propionate	3.4 ± 0.22	5.2 ± 0.22	<0.0001 ****
Valerate	0.45 ± 0.03	0.32 ± 0.02	0.0070 **
Isovalerate	0.099 ± 0.007	0.049 ± 0.005	<0.0001 ****
Glucose	0.51 ± 0.04	2.0 ± 0.19	<0.0001 ****
Glycerol	2.5 ± 0.80	0.31 ± 0.06	0.0015 **
Threonine	0.26 ± 0.02	0.19 ± 0.02	0.0355 *
Leucine	0.39 ± 0.05	0.38 ± 0.03	0.726
Methionine	0.13 ± 0.01	0.14 ± 0.01	0.763
Valine	0.46 ± 0.04	0.43 ± 0.03	0.536
Proline	0.36 ± 0.04	0.36 ± 0.03	0.981
Lactate	0.13 ± 0.03	0.071 ± 0.01	0.105
Glutamate	0.75 ± 0.05	0.77 ± 0.06	0.828
Ethanol	5.6 ± 0.74	5.9 ± 0.85	0.912
